# Patterns of care and follow-up care of patients with uveal melanoma in German-speaking countries: a multinational survey of the German Dermatologic Cooperative Oncology Group (DeCOG)

**DOI:** 10.1007/s00432-020-03450-0

**Published:** 2020-11-21

**Authors:** Theresa Steeb, Anja Wessely, Mareike Alter, Christiane Bayerl, Armin Bender, Guido Bruning, Evelyn Dabrowski, Dirk Debus, Nina Devereux, Edgar Dippel, Konstantin Drexler, Pia Dücker, Reinhard Dummer, Steffen Emmert, Peter Elsner, Alexander Enk, Christoffer Gebhardt, Anja Gesierich, Matthias Goebeler, Sergij Goerdt, Steven Goetze, Ralf Gutzmer, Sebastian Haferkamp, Gesina Hansel, Jessica C. Hassel, Lucie Heinzerling, Katharina C. Kähler, Kjell M. Kaume, Wolfgang Krapf, Nicole Kreuzberg, Percy Lehmann, Elisabeth Livingstone, Harald Löffler, Carmen Loquai, Cornelia Mauch, Johanna Mangana, Friedegund Meier, Markus Meissner, Rose K. C. Moritz, Lara Valeska Maul, Verena Müller, Peter Mohr, Alexander Navarini, Ahn Van Nguyen, Christiane Pfeiffer, Claudia Pföhler, Christian Posch, Erika Richtig, Rainer Rompel, Michael M. Sachse, Stefanie Sauder, Dirk Schadendorf, Kerstin Schatton, Hans-Joachim Schulze, Erwin Schultz, Bastian Schilling, Matthias Schmuth, Jan. C. Simon, Markus Streit, Patrick Terheyden, Alexander Thiem, Thomas Tüting, Julia Welzel, Gerhard Weyandt, Ulrich Wesselmann, Uwe Wollina, Mirjana Ziemer, Lisa Zimmer, Markus Zutt, Carola Berking, Max Schlaak, Markus V. Heppt, the German Dermatologic Cooperative Oncology Group (DeCOG, committee ocular melanoma)

**Affiliations:** 1 Department of Dermatology, University Hospital Erlangen, Friedrich-Alexander-University Erlangen-Nuremberg (FAU), Ulmenweg 18, 91054 Erlangen, Germany; 2 Comprehensive Cancer Center Erlangen, European Metropolitan Region of Nuremberg, Erlangen, Germany; 3 Department of Dermatology, University Medical Center, Magdeburg, Germany; 4 Department of Dermatology and Allergology, Skin Cancer Center Wiesbaden, Helios Dr. Horst Schmidt Clinics, Wiesbaden, Germany; 5 Department of Dermatology and Allergology, Philipps University Marburg, Marburg, Germany; 6 Center for Venous and Dermatosurgery, Tabea Krankenhaus Hamburg, Hamburg, Germany; 7 Department of Dermatology, Ludwigshafen Medical Center, Ludwigshafen, Germany; 8 Department of Dermatology, Paracelsus Medical University Nuremberg, City Hospital of Nuremberg, Nuremberg, Germany; 9 Department of Dermatology, University Hospital Regensburg, Regensburg, Germany; 10 Department of Dermatology, Hospital of Dortmund, Dortmund, Germany; 11 Department of Dermatology, University Hospital Zurich, University Zurich, Zürich, Switzerland; 12 Department of Dermatology and Venereology, University Medical Center, Rostock, Germany; 13 Department of Dermatology, University Hospital, Jena, Germany; 14 Department of Dermatology, University Hospital Heidelberg, Heidelberg, Germany; 15 Department of Dermatology and Venereology, University Medical Center Hamburg-Eppendorf, Hamburg, Germany; 16 Department of Dermatology, Venereology and Allergology, University Hospital Würzburg, Würzburg, Germany; 17 Department of Dermatology, Venerology and Allergology, Medical Faculty Mannheim, University Medical Center Mannheim, Heidelberg University, Mannheim, Germany; 18 Department of Dermatology and Allergy, Hannover Medical School, Skin Cancer Center Hannover, Hannover, Germany; 19 Department of Dermatology and Allergology, Städtisches Klinikum Dresden, Academic Teaching Hospital of the Technical University of Dresden, Dresden, Germany; 20 Department of Dermatology, Venereology and Allergology, University Medical Center of Schleswig-Holstein, Campus Kiel, Kiel, Germany; 21 Department of Dermatology and Allergology, Klinikum Bremen-Mitte, Bremen, Germany; 22 Department of Dermatology, SLK Hospital Heilbronn, Heilbronn, Germany; 23 Department of Dermatology and Venerology, University of Cologne, Cologne, Germany; 24 Department of Dermatology, Helios University Hospital Wuppertal, Wuppertal, Germany; 25 Department of Dermatology, University Hospital Essen, University Duisburg-Essen, Essen, Germany; 26 Department of Dermatology, University Medical Center Mainz, Mainz, Germany; 27 Department of Dermatology, University Hospital Dresden, Dresden, Germany; 28 Department of Dermatology, Venereology and Allergology, Johann Wolfgang Goethe University, Frankfurt/Main, Germany; 29 Department of Dermatology and Venereology, Martin-Luther-University Halle-Wittenberg, Halle (Saale), Germany; 30 Department of Dermatology, University Hospital Basel, Basel, Switzerland; 31 Department of Dermatology, Elbe-Kliniken, Buxtehude, Germany; 32 Department of Dermatology, Venerology and Allergology, Medical University of Innsbruck, Innsbruck, Austria; 33 Department of Dermatology and Allergology, University Hospital Augsburg, Augsburg, Germany; 34 Department of Dermatology and Allergology, University Hospital Ulm, Ulm, Germany; 35 Department of Dermatology, Saarland University Medical Center, Homburg/Saar, Germany; 36 Department of Dermatology and Allergy, School of Medicine, German Cancer Consortium (DKTK), Technical University of Munich, Munich, Germany; 37 Department of Dermatology, Medical University of Graz, Graz, Austria; 38 Department of Dermatology, Klinikum Kassel, Kassel, Germany; 39 Skin Cancer Center, Department of Dermatology, Allergology and Phlebology, Bremerhaven Reinkenheide, Bremerhaven, Germany; 40 Medical Faculty, Department of Dermatology, Heinrich-Heine-University, Düsseldorf, Germany; 41 Department of Dermatology and Dermato-Histo-Pathology, Fachklinik Hornheide, Skin Cancer Centre, Münster, Germany; 42 Department of Dermatology, Venereology and Allergology, University Medical Center Leipzig, Leipzig, Germany; 43 Department of Dermatology, Hospital Aarau, Aarau, Switzerland; 44 Department of Dermatology, University of Lübeck, Lübeck, Germany; 45 Department of Dermatology and Allergology, Hospital Bayreuth, Bayreuth, Germany; 46 Department of Dermatology and Allergy, University Hospital, Ludwig-Maximilians-University (LMU), Frauenlobstr. 9-11, 80337 Munich, Germany

**Keywords:** Uveal melanoma, Patterns of care, Cross-sectional study, Ocular melanoma, Surveillance, Follow-up, Treatment patterns, Background

## Abstract

**Purpose:**

Uveal melanoma (UM) is an orphan cancer of high unmet medical need. Current patterns of care and surveillance remain unclear as they are situated in an interdisciplinary setting.

**Methods:**

A questionnaire addressing the patterns of care and surveillance in the management of patients with uveal melanoma was distributed to 70 skin cancer centers in Austria, Germany and Switzerland. Frequency distributions of responses for each item of the questionnaire were calculated.

**Results:**

44 of 70 (62.9%) skin cancer centers completed the questionnaire. Thirty-nine hospitals were located in Germany (88.6%), three in Switzerland (6.8%) and two in Austria (4.5%). The majority (68.2%) represented university hospitals. Most patients with metastatic disease were treated in certified skin cancer centers (70.7%, 29/41). Besides, the majority of patients with UM were referred to the respective skin cancer center by ophthalmologists (87.2%, 34/39). Treatment and organization of follow-up of patients varied across the different centers. 35.1% (14/37) of the centers stated to not perform any screening measures.

**Conclusion:**

Treatment patterns of patients with uveal melanoma in Germany, Austria and Switzerland remain extremely heterogeneous. A guideline for the treatment and surveillance is urgently needed.

## Background

Ocular melanoma is a rare cancer condition that can develop as uveal or conjunctival tumors. Uveal melanoma (UM) represents one of the most common ocular malignancies among adults and accounts for about 5% of all melanoma cases. Primary tumors originate from the pigment cells of the choroid layer, the ciliary body or iris of the eye (Chattopadhyay et al. 2016). With an incidence of 4–7 cases per million in Europe, it is much rarer than cutaneous melanoma (Mallone et al. 2012). Therapeutic options of local disease include radiation therapies or surgical approaches like local resection and enucleation of the affected eye. Although these measures are highly effective to achieve local tumor control, up to 50% of patients develop distant metastases, which are mostly localized to liver and lungs (Bedikian 2006).

Risk for metastases strongly depends on monosomy 3 (Shields et al. 2011). Once UM becomes metastatic, the disease course is often aggressive and the prognosis is dismal with an average survival time of 1 year across all therapeutic regimens (Rantala et al. 2019). A combined approach with local treatment and combined immunotherapy has been employed with a median overall survival of 18 months in a small group of patients (Kirchberger et al. 2018). Thus, therapy options remain limited and have often been adopted from cutaneous melanoma, although these entities differ clinically and genetically (Heppt et al. 2017b). Few intervention studies have been published for UM and sound randomized controlled trials are lacking. Neither targeted therapy with MEK inhibitors nor immune checkpoint blockade (ICB) demonstrated significantly improvement of the prognosis of patients with UM (Heppt et al. 2017b; Steeb et al. 2018). Thus, creating a solid and uniform framework or guideline for evidence-based treatment decisions remains challenging.

The management of UM is subject to country-specific regulations. Currently, only a few international and consensus-based guidelines exist (Mathis et al. 2018; Nathan et al. 2015; Simpson et al. 2014; Weis et al. 2016), a German guideline is currently not yet available. Besides, the care of patients with UM is organized in an interdisciplinary setting, involving ophthalmologists, oncologists, interventional radiologists and dermato-oncologists. As current patterns of care are heterogeneous and the optimal management for patients with UM is yet to be determined, we performed a tri-national cross-sectional study to explore the current standard of care in German-speaking skin cancer centers.

## Methods

As no validated survey existed for the objective of our study, the questionnaire with a total of 15 items was developed *de-novo* based on our institutional experience. The explorative survey included questions in a multiple-choice format regarding various treatment approaches for primary and metastatic disease of ocular melanoma [i.e., UM and conjunctival melanoma (CM)], follow-up of patients, as well as items related to interdisciplinary cooperation. The questionnaire was pre-tested by independent researchers for clarity and comprehension. Based on their suggestions, the questionnaire was revised to its final form. The full questionnaire is available upon request. We encouraged the centers to also reply to our survey in case that they had not seen any patients with UM in 2018.

The paper-based questionnaire was distributed via mail to 70 skin cancer centers in Germany, Austria and Switzerland on 6 August 2019. A reminding letter was sent to all non-responders prolonging the initially stated period for response from 2 up to 4 months to increase the response rate. Contact information of the 70 participating centers (69 certified skin cancer centers and one uncertified center) were obtained via *OncoMap®* by *OnkoZert®*, an independent institute by the German Cancer Society which is responsible for the inspection and certification of cancer centers and oncology centers in Germany, Austria, and Switzerland (https://www.onkozert.de/). Participation was voluntary and each center was allowed to participate only once in the survey (cross-sectional design). Answered questionnaires could be sent back via E-Mail, Fax, or regular mail.

Frequency distributions of responses for each item were calculated and reported descriptively as absolute values and percentages (%). Quantitative variables were expressed as median with ranges. Subgroup differences were explored with Mood’s Median-Test. A *p*-value of < 0.05 was considered as significant. Statistical analyses were conducted with SPSS (IBM SPSS Statistics version 25, IBM Corporation).

## Results

### Characteristics of the participating centers

**Fig. 1:**
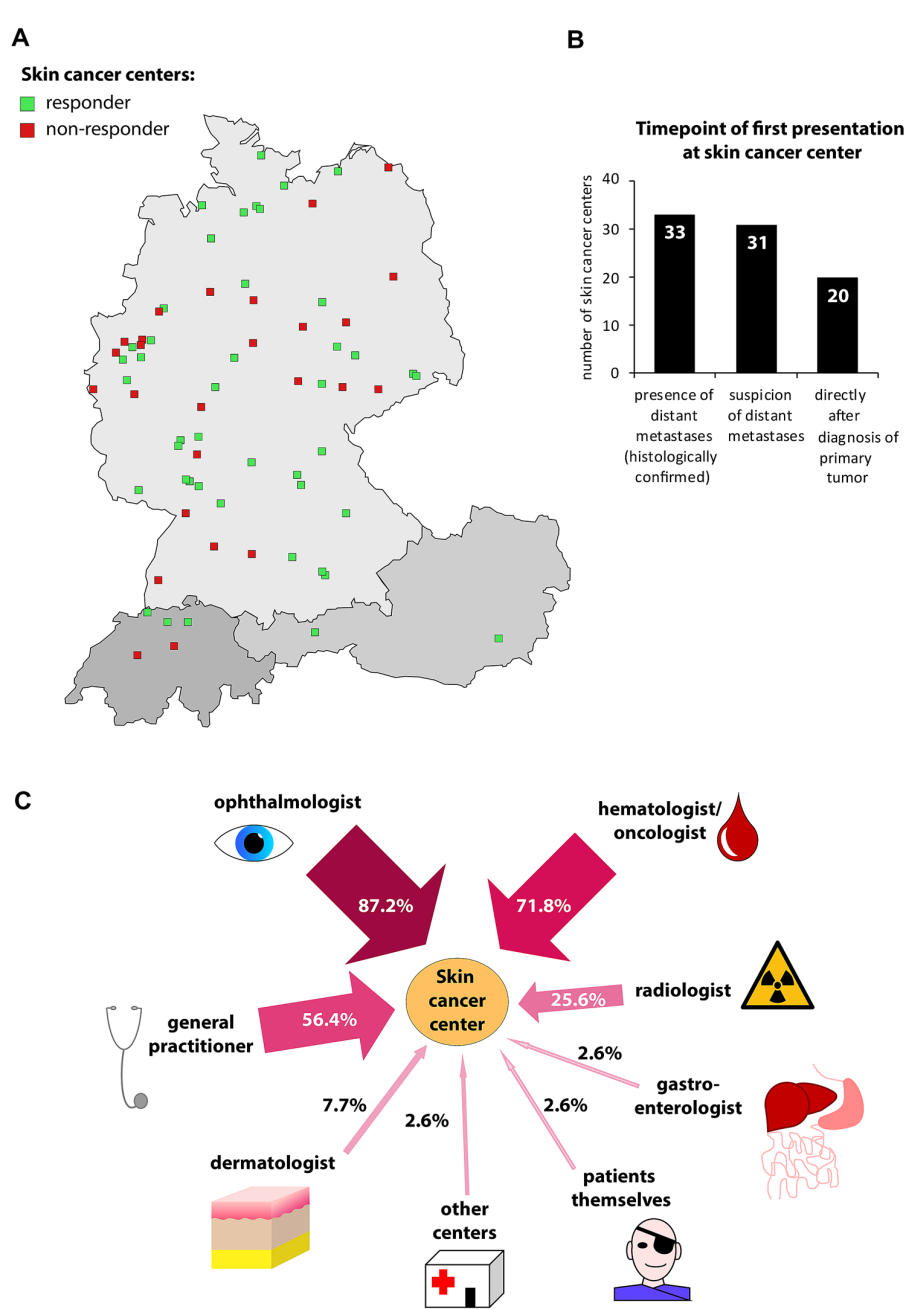
**a** Geographical map of the responding certified skin cancer centers in Austria, Germany, and Switzerland, **b** bar chart illustrating the timepoint of UM and CM patients’ first presentation to the skin cancer center, and **c** pattern of UM and CM patients’ referral by multidisciplinary professions to the skin cancer centers

Overall, 44 of 70 (62.9%) centers completed the questionnaire. Most of the responding centers were located in Germany (88.6%, *n* = 39), three were located in Switzerland (6.8%) and two in Austria (4.5%). The majority (68.2%, 30/44) represented university hospitals and one third municipal or private hospitals (31.8%, 14/44) (Fig. 1a).

In 86.8% (33/38) of the centers, patients with UM or CM presented for the first time after histological confirmation of distant metastases, followed by presentation upon clinical or radiological suspicion of distant metastases in 83.8% (31/38). In nearly half of the centers (52.6%, 20/38), UM or CM patients presented after treatment of the primary ocular tumor (Fig. 1b).

Patients with UM or CM were referred by ophthalmologists (87.2%, 34/39), hematologists/oncologists (71.8%, 28/39), general practitioners (56.4%, 22/39), and radiologists (25.6%, 10/39) (Fig. 1c). Other professions mentioned in the free-text field included dermatologists (*n* = 3), patients themselves (*n* = 1), gastroenterologists (*n* = 1) or others (*n* = 3).

### Surveillance

A total of 460 patients with primary UM were estimated to be treated at the skin cancer centers in 2018. 396 of these 460 patients with primary UM (86.1%) were treated in German skin cancer centers. The number of patients undergoing surveillance for UM at the skin cancer centers in 2018 ranged from 0 to 100 (median 3). Overall, 11.4% (5/44) of the participating centers did not have any patients in surveillance. Notably, more patients were followed-up on in a university hospital setting than in municipal or private hospitals (*p* = 0.013).

### Surveillance of uveal melanoma (stages I–IIIC)

Half of the centers (51.4%, 19/37) performed regular screening measures to detect metastatic disease after the primary diagnosis of UM in case of a high-risk profile, such as large primary tumor or high vertical tumor thickness. Screening due to a high molecular risk profile such as monosomy 3 was performed in 32.4% (12/37). In contrast, 35.1% (14/37) did not perform any screening measures. The surveillance was exclusively performed by ophthalmology departments in three centers.

**Fig. 2:**
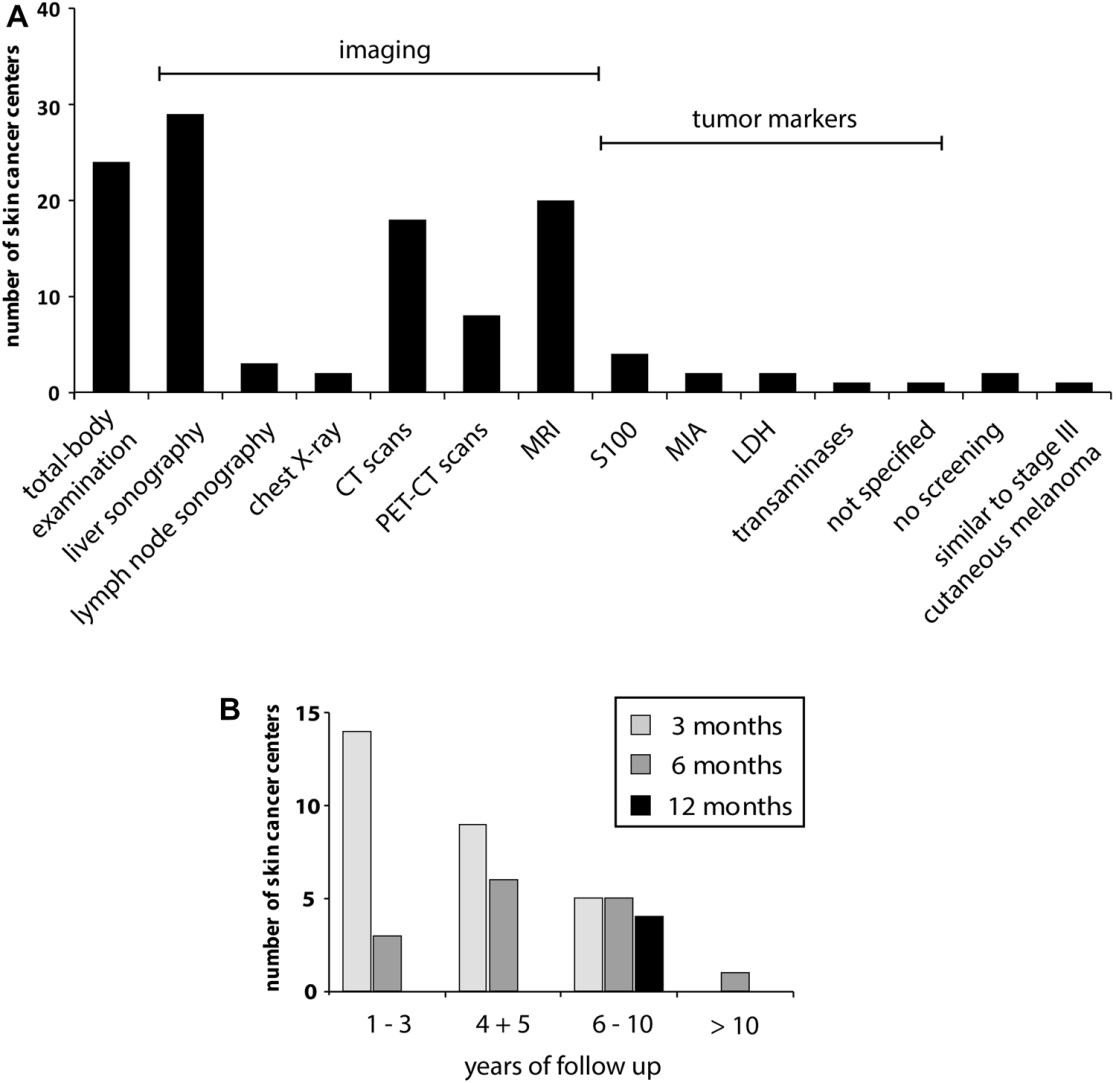
Bar chart illustrating **a** the deployed screening measures in the skin cancer centers for patients with UM **b** applied screening intervals in different years of follow-up for UM. Abbreviations: CT: computed tomography, PET-CT: positron emission tomography–computed tomography, MRI: magnetic resonance imaging, MIA: melanoma inhibitory antigen, LDH: lactate dehydrogenase

When asked about the specific screening and diagnostic measures performed during follow-up care, 76.3% (29/38) reported liver sonography, followed by total-body examination (63.2%, 24/38), magnetic resonance imaging (MRI) (52.6%, 20/38), computed tomography (CT) scans (47.4%, 18/38) and positron emission tomography–computed tomography (PET-CT) (21.1%, 8/38) (Fig. 2a). Other modalities were lymph node sonography (*n* = 3) and chest x-ray (*n* = 2).

Other screening measures stated in a free-text field comprised the assessment of serum biomarkers including S100 (*n* = 4), melanoma inhibitory activity protein (MIA, *n* = 2), liver enzymes (*n* = 1), lactate dehydrogenase (LDH, *n* = 2) or tumor markers in general (*n* = 1). One center had no uniform guidelines but performed regular sonography or MRI of the liver. Another one adapted the recommendations for the follow-up of stage III cutaneous melanoma.

The majority performed follow-up examinations at intervals of 3 months (66.7%, 22/33) or 6 months (51.5%, 17/33). Besides, three centers conducted surveillance at intervals of 12 months (9.1%), while one stated that intervals were longer than 12 months. No specific intervals were reported by three centers. Twenty-one centers provided more information about the screening intervals in different years of follow-up (Fig. 2b). The screening intervals differed between centers especially when the time of follow-up was longer than 3 years. Twelve centers also provided detailed information about distinct screening measurements and intervals (Table 1).

**Table 1:**
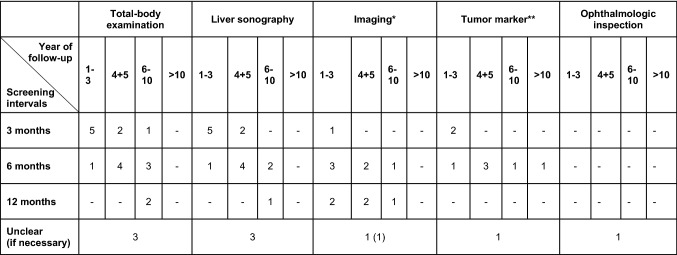
Reported follow-up strategies for UM in the skin cancer centers in Austria, Germany, and Switzerland

*MRI, CT, PET-CT, chest X-ray

**S100, LDH, MIA, liver enzymes

### Management of metastatic disease

Overall, 249 patients with metastatic UM (median: 3, range 0–50) were treated in 2018 in the participating centers. The median significantly differed between university and municipal hospitals (*p* = 0.003), i.e., more patients with metastatic disease were followed-up on in university hospitals.

Most patients with metastatic disease (UM and CM) were treated in the skin cancer centers or dermatology departments (70.7%, 29/41), while 12.2% (5/41) indicated that patients were treated in hematology/oncology units only. In 5 centers (12.2%), patients with metastatic disease were treated both by skin cancer centers and hematology/oncology units. One center reported simultaneous treatment in the ophthalmology and gastroenterology departments, respectively. In one center, patients with metastatic disease were treated in the gastroenterology department.

**Fig. 3:**
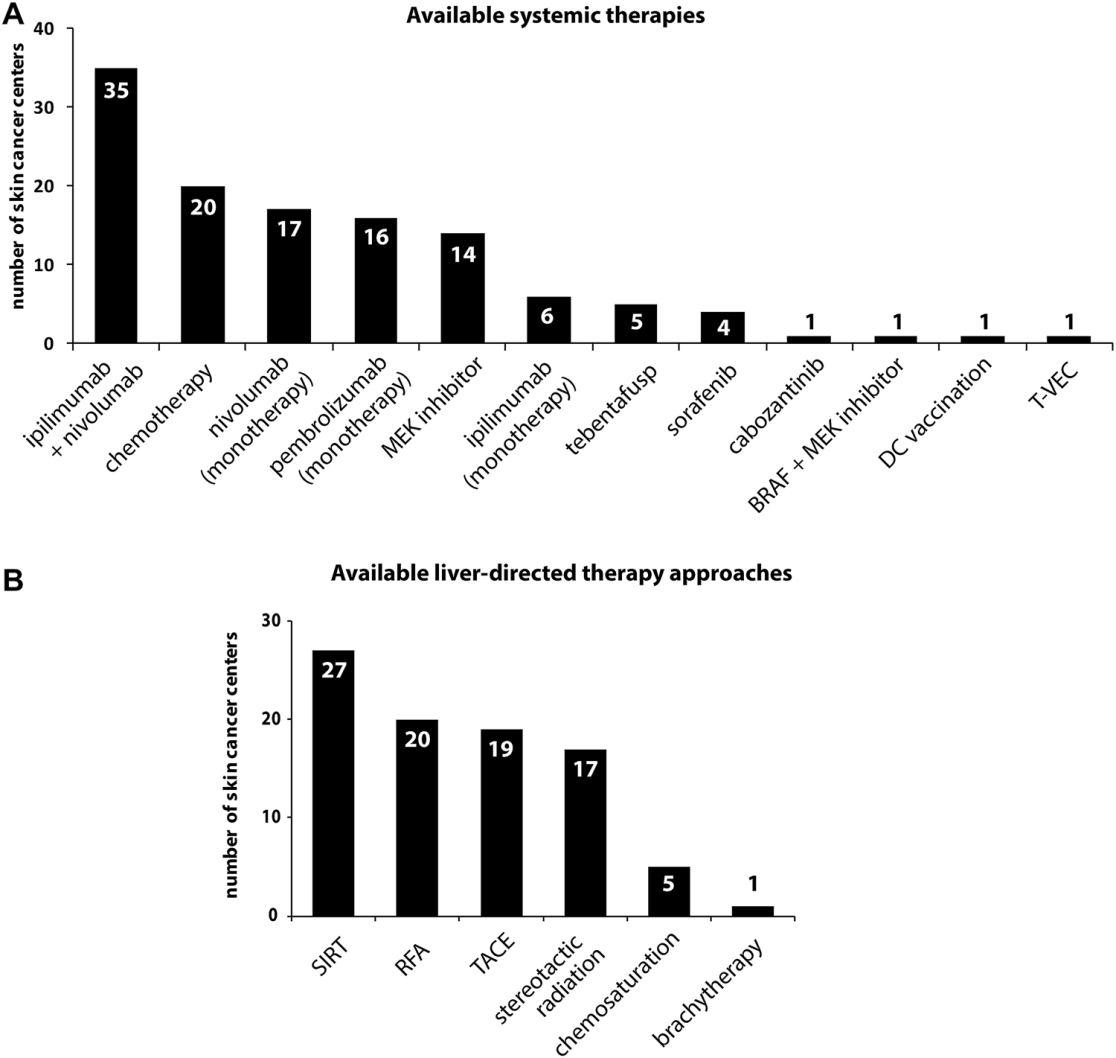
Bar charts illustrating **a** available systemic treatments in the skin cancer centers and **b** liver-directed approaches for the management of metastatic UM and CM. Abbreviations: DC: dendritic cell, T-VEC: talimogene laherparepvec, SIRT: selective internal radiation therapy, RFA: radiofrequency ablation, TACE: trans-arterial chemoembolization

We also investigated which systemic treatments were applied for metastatic UM in 2018. Nearly 80% of centers (35/40) applied nivolumab in combination with ipilimumab, followed by conventional chemotherapy (50%, 20/40), nivolumab monotherapy (42.5%, 17/40), and pembrolizumab monotherapy (40%, 16/40) (Fig. 3a). Besides, MEK inhibitors and ipilimumab monotherapy were applied in 35% (14/40) and 15% (6/40), respectively. Other treatments mentioned in a free-text field included tebentafusp (*n* = 5), sorafenib (*n* = 4) and cabozantinib (*n* = 1), BRAF plus MEK inhibitor (*n* = 1), dendritic cell vaccination (*n* = 1), and talimogene laherparepvec (*n* = 1).

As UM has a unique propensity for metastatic spread to the liver, we specifically investigated liver-directed treatments (Collaborative Ocular Melanoma Study 2001). Selective internal radiation therapy (SIRT) was most frequently performed (79.4%; 27/34), followed by radiofrequency ablation (RFA) with 58.8% (20/34), trans-arterial chemoembolization (TACE) (55.9%, 19/34), and stereotactic radiation (50%, 17/34) (Fig. 3b).

### Implications and unmet needs

At the end of the questionnaire, all responders were invited to raise general comments or wishes regarding the management of UM. Most desired clear follow-up schemes for the surveillance of tumor-free patients with UM. Surveillance, especially regarding intervals and duration of follow-up, is currently not yet standardized, but rather individual. Guidelines are, therefore, desirable as currently treatment of patients with UM is mostly based on individual experience. Additionally, a central register for patients with ocular melanoma was suggested by one center.

## Discussion

Uveal melanoma is a rare cancer condition of high unmet clinical need. The aim of our survey was to provide an overview of the current patterns of care and surveillance in German-speaking skin cancer centers. We focused on skin cancer centers as the treatment of metastatic disease is currently mostly performed in analogy to cutaneous melanoma including checkpoint blockade and kinase inhibitors. Not surprisingly, more patients were treated in follow-up care at university hospitals compared to municipal hospitals. In 2016, 642 adult patients were newly diagnosed with ocular cancer (ICD-10 C69) in Germany, indicating that the skin cancer centers represented in our survey treated approximately more than 70% of all newly diagnosed cases (Robert Koch-Institut 2020). Taking into account that up to 50% of all patients with UM develop distant metastases (Bedikian 2006) approximately 320 patients in Germany will be diagnosed with metastatic disease per year. In our survey, the skin cancer centers stated to care for 253 patients, indicating that in Germany, almost 80% of all patients with metastatic ocular melanoma, i.e., UM or CM, are treated in these departments. These numbers highlight the importance of skin cancer centers for the therapy and surveillance of patients with both primary and metastatic ocular melanoma.

Although UM differs from cutaneous melanoma both clinically and biologically, treatment options for advanced stages have largely been adopted from it, yet with much lower response rates and at the cost of high treatment-related toxicity (Heppt et al. 2017a, 2019). This makes it difficult to demonstrate the clinical effectiveness of interventions and to create a solid framework for evidence-based treatment decisions. Our survey among skin cancer centers in Germany, Austria and Switzerland confirmed the urgent need for the development of clinical practice guidelines for this rare cancer entity, comparable to other consortia (Mathis et al. 2018; Nathan et al. 2015; Simpson et al. 2014; Weis et al. 2016). Special strategies need to be employed to generate evidence that is compatible with rigorous quality standards of guidelines (Pai et al. 2019). Previous assessments of the methodological quality of international guidelines on UM have identified weaknesses and strengths of existing guidelines which require particular attention and improvement in future guidelines (Steeb et al. 2020). A guideline from the United Kingdom published by Nathan et al. was rated as best and may hence serve as a basis for a future German guideline.

A further barrier towards the management of UM comes from the fact that the care of patients with UM occurs in a highly interdisciplinary setting, involving ophthalmologists, hematologists, oncologists, interventional radiologists and dermato-oncologists. This highlights the urgent need for an interdisciplinary guideline. For patients with primary disease, mostly ophthalmologists and radiation oncologists are involved in the care, while patients with metastatic disease are often referred to dermato-oncologists, which is in line with our sample. The majority of surveyed responders stated that patients presented to their center after the histological confirmation of distant metastases. Thus, ICB is often adopted as therapeutic strategy from cutaneous melanoma with 80% of centers treating metastatic patients with nivolumab plus ipilimumab. Beside the broad usage of ICB, chemotherapy was also often applied although it has only limited efficacy irrespective of the chemotherapeutic agent (Carvajal et al. 2017). This underlines that novel treatment strategies are utterly needed. Clinical studies investigating new treatment options as, e.g., tebentafusp (IMCgp100), a bispecific protein bridging CD3 ^+^  T cells and gp100-expressing tumor cells (Liddy et al. 2012) have shown promising results in phase I/II trials so far (Carvajal et al. 2018; Middleton et al. 2016).

Screening and follow-up of patients varied the most across our sample. Some followed the evidence- and consensus-based guideline for stage III cutaneous melanoma (Eigentler et al. 2017), while others based their follow-up schedule on the recommendations established within their institution or on personal experience. This highlights again the urgent need for uniform recommendations which should be based on progression and recurrence rate.

There are several limitations of this study. The response rate was limited to 44 of 70 skin cancer centers. Besides this, university hospitals were overrepresented, thus diminishing the overall representability of the results. Furthermore, recall bias may be likely as the participating centers had to remember management of patients of the years before. Additionally, in some items, the answers of the participants differed extremely for instance when reporting the follow-up schemes, ranging from only brief descriptions to detailed reports including follow-up intervals and screening methods. However, to the best of our knowledge, practice and surveillance patterns in German-speaking countries have not been investigated so far, and therefore, our results represent a first step towards standardized care for patients with ocular melanoma.

Our results will contribute to improve nationwide management of ocular melanoma and to deduce possible future projects within the German Dermatologic Cooperative Oncology Group (DeCOG).

## Availability of data and material

The data that support the findings of this study are available on request from the corresponding author. The data are not publicly available due to privacy restrictions.

## Data Availability

SPSS Statistics version 25 (IBM Corporation, Armonk, NY, USA) was used to analyze the data.
